# Supplementary material for: Skin transcriptional profiles in Oophaga poison frogs
Source: Genet Mol Biol. 2020 Nov 16;43(4):e20190401. doi: 10.1590/1678-4685-GMB-2019-0401 (PMC7678260; doi:10.1590/1678-4685-GMB-2019-0401)
Supplement: Supplementary file 1 [file 1415-4757-GMB-43-4-e20190401-s1.pdf]

Genetics and Molecular Biology
https://doi.org/10.1590/1678-4685-GMB-2019-0401

Supplementary Material to "Skin transcriptional profiles in *Oophaga* poison frogs"

Table S1 - Number of reads and library type (i.e paired vs. single-end) for each individual NA-seq experiment.

Individual library	Raw number of reads	Reads after filtering	Remaining % of reads	Library type
O. solanensis	128,599,724	105,179,065	81%	Paired reads*
O. anchicayensis	124,232,804	110,217,128	88%	
O. lehmanni	102,225,680	91,493,848	89%	
O. sylvatica	48,929,329	45,147,654	92%	Single reads
O. lehmanni	43,633,080	41,086,379	94%	
***O. anchicayensis*)**	7,998,636	7,173,285	89%	
O. histrionica	50,204,617	47,376,912	94%	
**Reference transcriptome**	N/A	33,895,474	100%	Single reads

*. Number of reads correspond to the combined value of both ends libraries.
