# Supplementary material for: Skin transcriptional profiles in Oophaga poison frogs
Source: Genet Mol Biol. 2020 Nov 16;43(4):e20190401. doi: 10.1590/1678-4685-GMB-2019-0401 (PMC7678260; doi:10.1590/1678-4685-GMB-2019-0401)
Supplement: Supplementary file 2 [file 1415-4757-GMB-43-4-e20190401-s2.pdf]

Genetics and Molecular Biology
https://doi.org/10.1590/1678-4685-GMB-2019-0401

Supplementary Material to "Skin transcriptional profiles in *Oophaga* poison frogs"

Table S2 - RNA-sequence datasets from cryptic species used for the differential expression analysis of this work.

Species	Experiment (SRA*)	RNA-seq library	Total bases	Date available
Bufo gargarizans	SRX2640691	Paired-end (150 b)	19.2 Gbp	2017-03-15
Pelophylax nigromaculatus	SRX2640690	Paired-end (150 b)	18.0 Gbp	2017-03-15
Polypedates megacephalus	SRX1720193	Paired-end (100 b)	6.3 Gbp	2016-04-25
Rana catesbeiana	SRX2988982	Paired-end (100 b)	9.2 Gbp	2017-07-07
Rana sylvatica	SRX2989023	Paired-end (100 b)	8.9 Gbp	2017-07-07
Xenophrys sangzhiensis	SRX1720191	Paired-end (100 b)	6.8 Gbp	2016-04-25

* Sequence Read Archive accession number from Genbank.
