# Supplementary material for: Skin transcriptional profiles in Oophaga poison frogs
Source: Genet Mol Biol. 2020 Nov 16;43(4):e20190401. doi: 10.1590/1678-4685-GMB-2019-0401 (PMC7678260; doi:10.1590/1678-4685-GMB-2019-0401)
Supplement: Supplementary file 3 [file 1415-4757-GMB-43-4-e20190401-s6.pdf]

Genetics and Molecular Biology
https://doi.org/10.1590/1678-4685-GMB-2019-0401

Supplementary Material to "Skin transcriptional profiles in *Oophaga* poison frogs"

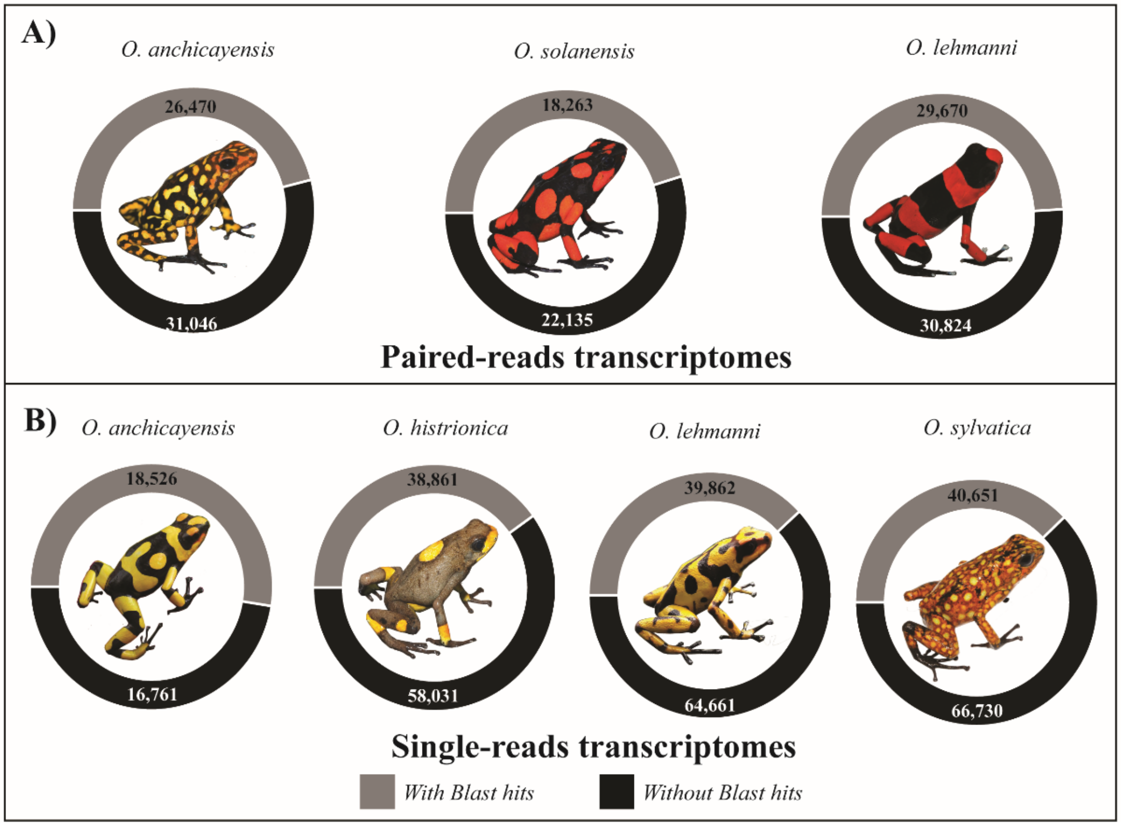

Figure S1 - Pie charts representing the number and proportion of contigs with significant *BLAST* hits (E<1.0E^{-5}) for each individual transcriptome from *Oophaga* species. Transcriptomes obtained using paired-ends RNA-seq libraries are represented in (A) and those obtained with single-end libraries in (A).
