# Supplementary material for: Skin transcriptional profiles in Oophaga poison frogs
Source: Genet Mol Biol. 2020 Nov 16;43(4):e20190401. doi: 10.1590/1678-4685-GMB-2019-0401 (PMC7678260; doi:10.1590/1678-4685-GMB-2019-0401)
Supplement: Supplementary file 4 [file 1415-4757-GMB-43-4-e20190401-s3.pdf]

Genetics and Molecular Biology
https://doi.org/10.1590/1678-4685-GMB-2019-0401

Supplementary Material to "Skin transcriptional profiles in *Oophaga* poison frogs"

Table S3 - Quality assessment report for each individual and the composite reference transcriptome based on the protein count of the core eukaryotic genes (BUSCO, see methods in the main article).

**Transcriptome**	**Library**	**Busco v.2 mapping**		
		**Complete**	**Partial**	**Complete + Partial**
O. solanensis	Paired	20.60%	30.47%	51.07%
O. anchicayensis	Paired	24.46%	37.77%	62.23%
O. lehmanni	Paired	29.61%	40.77%	70.38%
O. sylvatica	Single	45.92%	33.48%	79.40%
O. lehmanni	Single	33.91%	39.91%	73.82%
O. anchicayensis	Single	9.87%	30.47%	40.34%
O. histrionica	Single	39.06%	37.77%	76.83%
**Reference assembly**	Single	45.92%	42.62%	88.54%
