# Supplementary material for: Skin transcriptional profiles in Oophaga poison frogs
Source: Genet Mol Biol. 2020 Nov 16;43(4):e20190401. doi: 10.1590/1678-4685-GMB-2019-0401 (PMC7678260; doi:10.1590/1678-4685-GMB-2019-0401)
Supplement: Supplementary file 5 [file 1415-4757-GMB-43-4-e20190401-s7.pdf]

Genetics and Molecular Biology
https://doi.org/10.1590/1678-4685-GMB-2019-0401

Supplementary Material to "Skin transcriptional profiles in *Oophaga* poison frogs"

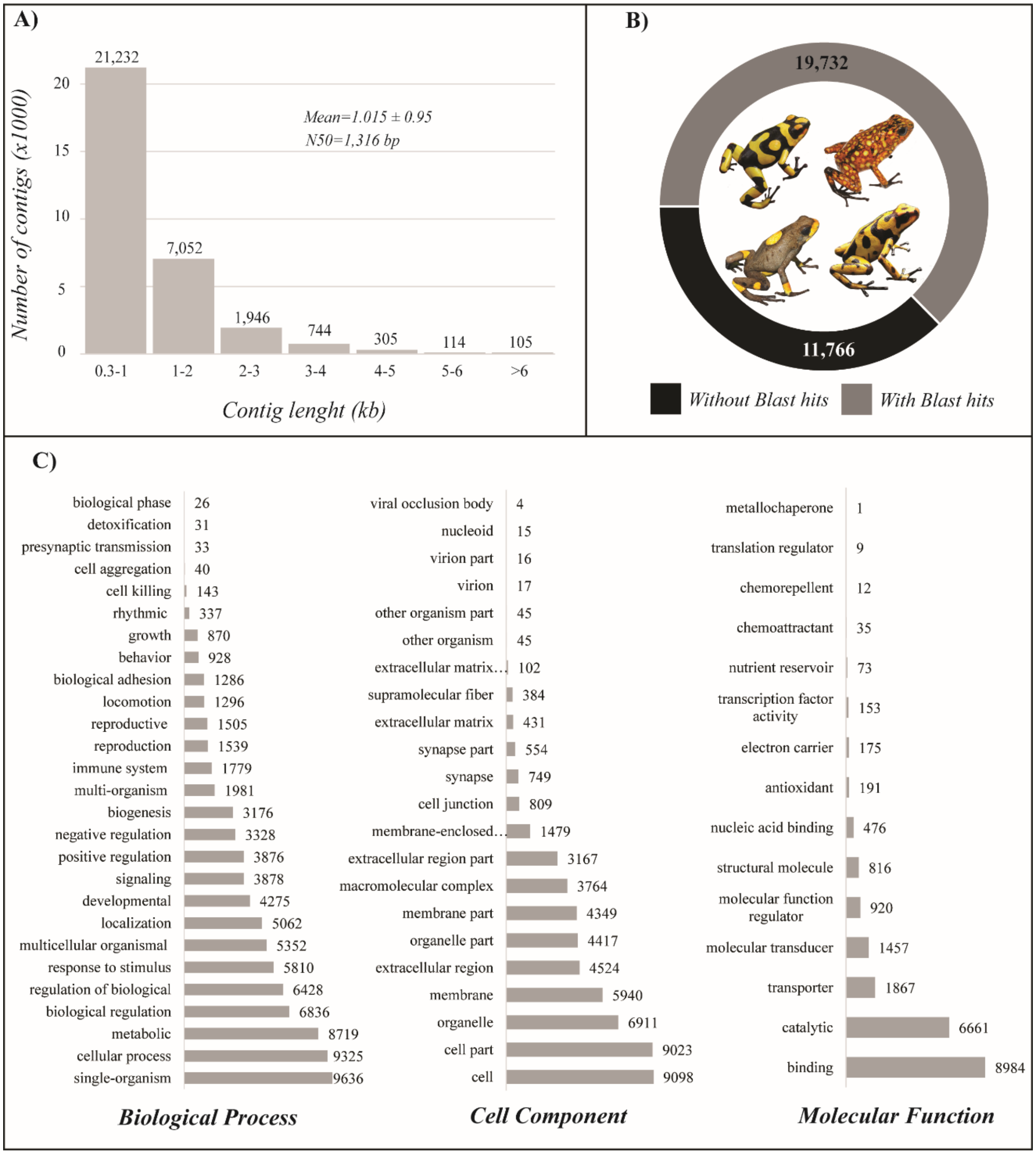

Figure S2 - A) Contig length distribution of the *de-novo* composite reference transcriptome of *Oophaga* species from skin tissue. B) Pie chart representing the number and proportion of contigs with significant *BLAST* hits (E<1.0E^{-5}) in the reference *Oophaga* transcriptome. C) Gene ontology (GO) categories distribution (level II) for the annotated unigenes in the *Oophaga* reference transcriptome.
