# Supplementary material for: Skin transcriptional profiles in Oophaga poison frogs
Source: Genet Mol Biol. 2020 Nov 16;43(4):e20190401. doi: 10.1590/1678-4685-GMB-2019-0401 (PMC7678260; doi:10.1590/1678-4685-GMB-2019-0401)
Supplement: Supplementary file 7 [file 1415-4757-GMB-43-4-e20190401-s9.pdf]

Genetics and Molecular Biology
https://doi.org/10.1590/1678-4685-GMB-2019-0401

Supplementary Material to "Skin transcriptional profiles in *Oophaga* poison frogs"

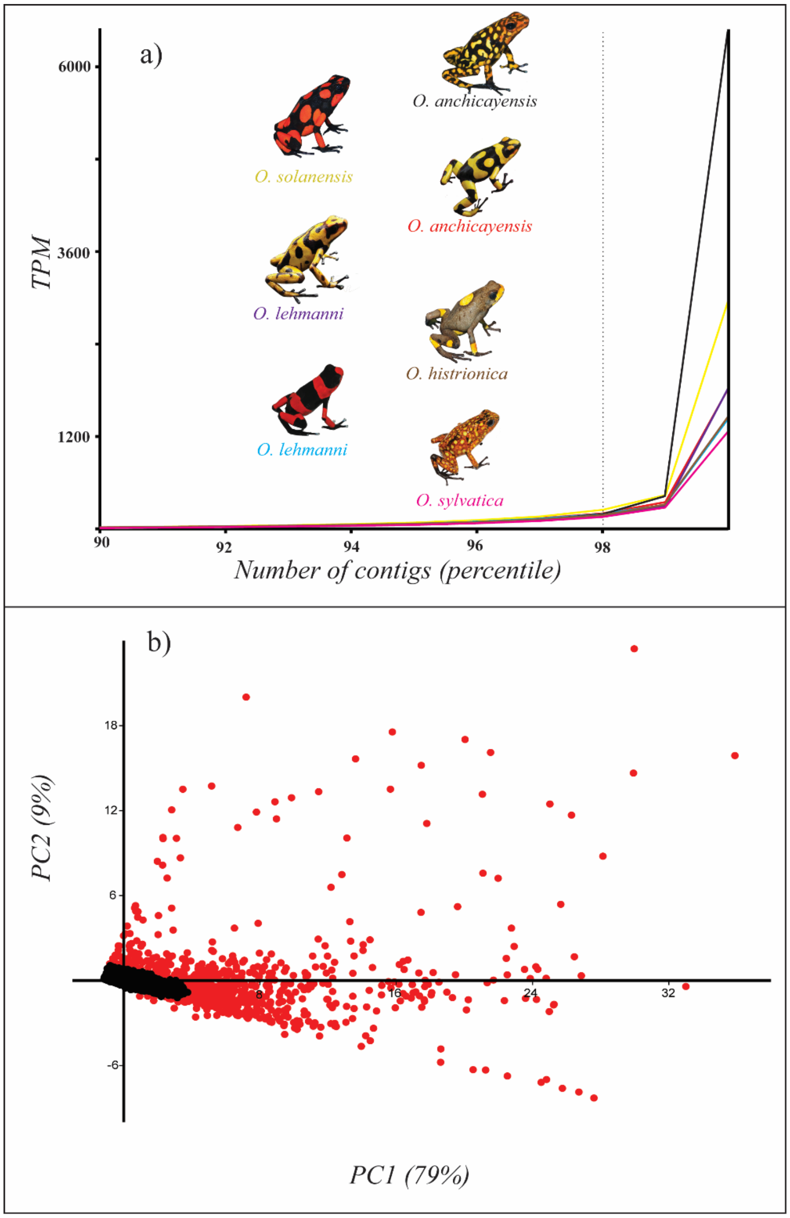

Figure S4 - A) Percentile plot of the estimated transcripts per million (TPM) in the composite reference transcriptome as calculated based on raw RNA reads from individual libraries. Color names are equivalent to those in tendency lines. B) Principal component analyses (PCA) plot of the reference contigs dataset using TPMs values as independent variables. Red dots represent the selected highly represented unigenes (2% of the total contigs; n= 1,437) while dark dots represent the remaining ones (n=30,061)
