# Supplementary material for: Skin transcriptional profiles in Oophaga poison frogs
Source: Genet Mol Biol. 2020 Nov 16;43(4):e20190401. doi: 10.1590/1678-4685-GMB-2019-0401 (PMC7678260; doi:10.1590/1678-4685-GMB-2019-0401)
Supplement: Supplementary file 8 [file 1415-4757-GMB-43-4-e20190401-s4.pdf]

Genetics and Molecular Biology
https://doi.org/10.1590/1678-4685-GMB-2019-0401

Supplementary Material to "Skin transcriptional profiles in *Oophaga* poison frogs"

Table S4 - Summary statistics of the differentially expressed unigenes (see methods) and their *BLAST* annotations.

	**Differentially expressed unigenes**	**With annotation**	**Without annotation**
**Up-regulated**	1656 (85.6%)	996 (51.6%)	660 (34.2%)
**Down-regulated**	275 (14.2%)	256 (13.3%)	19 (1%)
**Total**	1931 (100%)	1252 (64.9%)	679 (35.2%)
