# Supplementary material for: Skin transcriptional profiles in Oophaga poison frogs
Source: Genet Mol Biol. 2020 Nov 16;43(4):e20190401. doi: 10.1590/1678-4685-GMB-2019-0401 (PMC7678260; doi:10.1590/1678-4685-GMB-2019-0401)
Supplement: Supplementary file 9 [file 1415-4757-GMB-43-4-e20190401-s10.pdf]

Genetics and Molecular Biology
https://doi.org/10.1590/1678-4685-GMB-2019-0401

Supplementary Material to "Skin transcriptional profiles in *Oophaga* poison frogs"

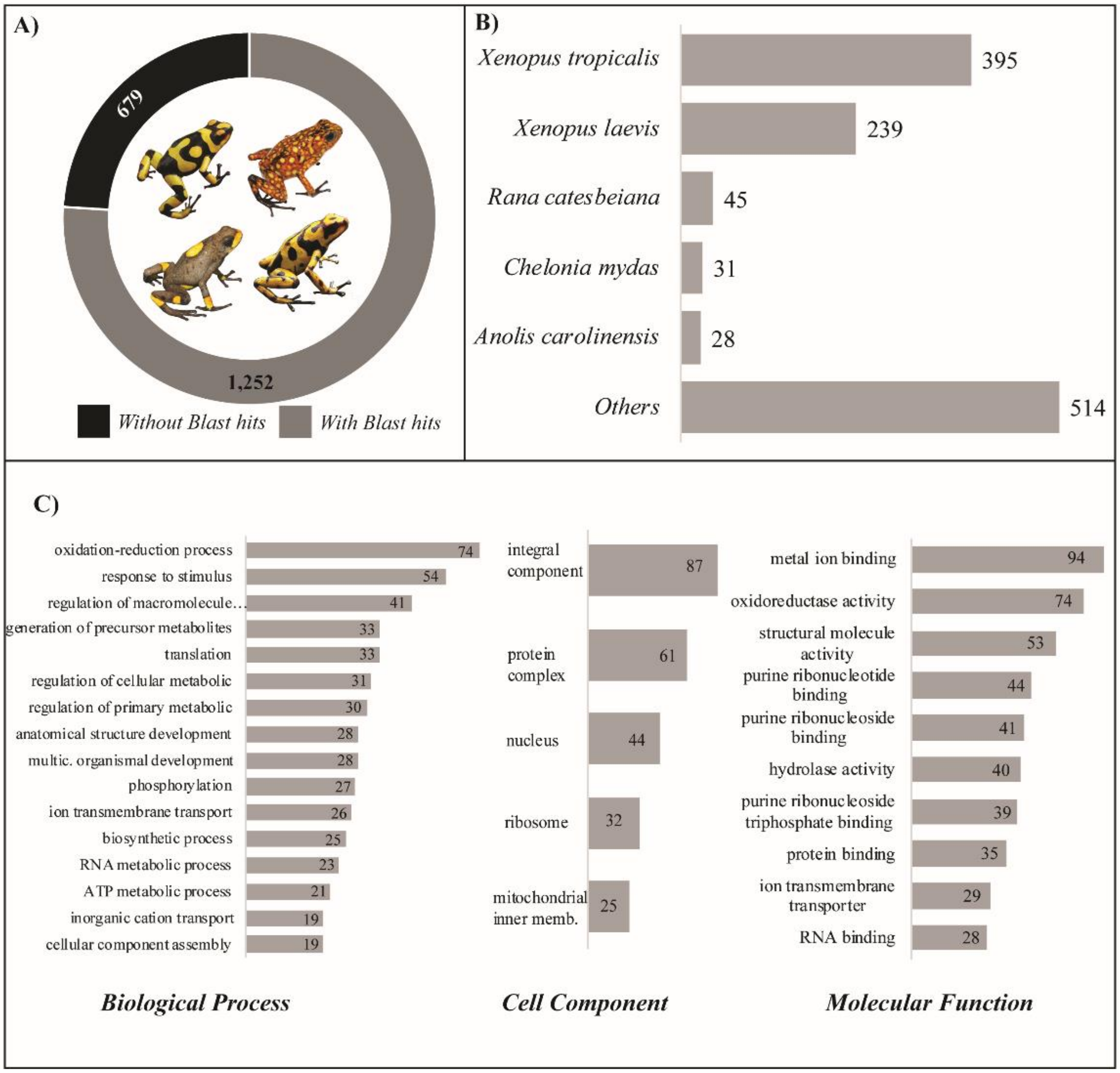

Figure S5 - A) Pie chart representing the total number of differentially expressed unigenes and the proportion with significant *BLAST* hits ($E<1.0E^{-5}$). B) BLAST hit species distribution of highly expressed unigenes. C) Gene ontology (GO) categories distribution (multilevel) for the annotated highly expressed unigenes.
