# Supplementary material for: Skin transcriptional profiles in Oophaga poison frogs
Source: Genet Mol Biol. 2020 Nov 16;43(4):e20190401. doi: 10.1590/1678-4685-GMB-2019-0401 (PMC7678260; doi:10.1590/1678-4685-GMB-2019-0401)
Supplement: Supplementary file 10 [file 1415-4757-GMB-43-4-e20190401-s5.pdf]

Genetics and Molecular Biology
https://doi.org/10.1590/1678-4685-GMB-2019-0401

Supplementary Material to "Skin transcriptional profiles in *Oophaga* poison frogs"

Table S5 - Transcripts with potential important functions in the alkaloid sequestration system, auto-resistance to toxic compounds and variation in coloration in harlequin poison frogs. The corresponding composite *Oophaga* transcriptome will be deposited at the Genbank database after acceptance.

Transcript ID	Fold change	padj	BLAST description	Length (bp)	GO Names list	
14256	779.11	0.00	Transmembrane protein 41B	1253	C:cell; P:gonad development; P:thyroid gland development; P:luteinizing hormone secretion; P:thyroid hormone generation; P:cellular response to hormone stimulus; P:follicle-stimulating hormone secretion; P:negative regulation of organ growth; C:extracellular region; F:hormone activity	Alkaloid transportation
26699	517.76	0.00	Transmembrane protein 181	1079	C:extracellular space	
14644	297.83	0.00	Transmembrane protein 116-like	1743		
14399	192.77	0.01	Transmembrane protein 26	1039		
6476	174.07	0.00	Transmembrane protein	1690	F:structural constituent of eye lens	

15428	105.09	0.02	Transmembrane protein 220	697	F:chondroitin sulfate binding; C:nucleus; F:identical protein binding; F:calcium-dependent protein binding; C:cytosol; C:chromaffin granule membrane; P:regulation of transcription, DNA-templated; F:heparin binding; P:negative regulation of cellular process; F:carbohydrate binding; C:apical plasma membrane	
8646	100.29	0.01	Transmembrane protein 69	936		
20904	66.89	0.04	Transmembrane protein 186-like	615		
19857	53.30	0.05	Transmembrane protein 70, mitochondrial	532	P:'de novo' pyrimidine nucleobase biosynthetic process; F:orotidine-5'-phosphate decarboxylase activity; P:'de novo' UMP biosynthetic process	
24171	45.71	0.01	Transmembrane protein 125-like	4098		
19808	37.86	0.04	Keratinocyte-associated Transmembrane protein 2-like	1043		
16724	27.39	0.02	Transmembrane protein 206	1589	P:negative regulation of potassium ion transmembrane transport; P:positive regulation of myofibroblast contraction; P:response to salt; P:negative regulation of platelet aggregation; P:response to progesterone; P:positive regulation of vasoconstriction; P:negative regulation of gastrin-induced gastric acid secretion; C:neuronal cell body; P:negative regulation of insulin secretion; F:prostaglandin ….	
24522	25.75	0.00	Transmembrane protease serine 13-like	2292	F:oxidoreductase activity	

25621	7.42	0.05	Cation-transporting ATPase 13A4	469	P:response to dehydroepiandrosterone; F:gamma-catenin binding; C:extracellular space; P:response to 11-deoxycorticosterone; C:membrane; P:metabolic process; P:response to progesterone; P:response to estradiol; C:desmosome; F:hydrolase activity	
9463	-6.16	0.04	Lysosomal-trafficking regulator	850	F:calcium ion binding; P:keratinization	
15350	175.29	0.00	EF-hand calcium-binding domain-containing 10-like	436	F:calcium ion binding; C:cellular_component	Alkaloid auto-resistance: target -site insensitivity
16951	95.46	0.01	S100 calcium binding protein Z	2161	P:single-organism cellular localization; P:cell cycle process; F:binding; P:organelle organization; C:intracellular organelle part; P:single-organism transport; P:establishment of protein localization; C:endosome; C:membrane-bounded vesicle; P:vesicle-mediated transport; P:regulation of cellular process; C:membrane; C:cytoplasmic vesicle; P:establishment of localization in cell; P:cellular protein localization; P:establishment of organelle localization	
1216	63.08	0.03	Extracellular calcium-sensing receptor-like	1214	C:extracellular space; F:carboxypeptidase activity; P:bradykinin catabolic process; P:proteolysis; P:response to glucocorticoid	
18107	4239.03	0.00	Potassium Voltage-gated channel subfamily E member 3-like	1864	P:ion transport; P:regulation of membrane potential; C:integral component of membrane; F:extracellular ligand-gated ion channel activity; P:synaptic transmission; C:synapse; F:binding;	

					P:regulation of biological process; P:neurological system process; P:response to stimulus; C:plasma membrane part	
16614	9.90	0.04	MGC114646 protein	1514	F:high voltage-gated calcium channel activity; F:protein C-terminus binding; C:voltage-gated calcium channel complex; P:calcium ion transmembrane transport; C:cytosol; P:positive regulation of cytosolic calcium ion concentration	
907	-6.46	0.01	Voltage-dependent anion channel 2	990		
376	-10.41	0.00	Voltage-dependent anion channel	388		
8966	102.76	0.01	Sodium channel, voltage-dependent, gamma subunit 1	640	C:sarcoplasmic reticulum; C:voltage-gated calcium channel complex; F:calcium channel regulator activity; F:voltage-gated calcium channel activity; F:mating pheromone activity; P:pheromone-dependent signal transduction involved in conjugation with cellular fusion; C:T-tubule; C:extracellular exosome; F:metal ion binding; P:mating; P:regulation of ion transmembrane transport; P:calcium ion transmembrane transport; P:regulation of calcium ion transport	

Fold change	padj	BLAST description	Length (bp)	GO Names list	
420.10	0.04	Cytochrome P450-like TBP	408		Alkaloid auto-resistance: metabolic detoxification
36.47	0.01	Cytochrome P450, family 4, subfamily F, polypeptide 22	458	F:oxygen binding; P:oxygen transport; C:voltage-gated potassium channel complex; F:delayed rectifier potassium channel activity; F:A-type (transient outward) potassium channel activity; P:protein homooligomerization; C:neuronal cell body; P:membrane repolarization; F:oxygen transporter activity; F:ion channel binding; F:heme binding; C:hemoglobin complex; C:dendrite; C:caveola; C:perinuclear endoplasmic reticulum; F:iron ion binding; P:cellular response to BMP stimulus; C:sarcolemma; P:regulation of ion transmembrane transport; P:potassium ion export	
30.72	0.02	Cytochrome P450 2F2-like	1201		
17.97	0.00	Cytochrome P450 2D26-like	1851	P:sucrose biosynthetic process; C:chromosome; F:binding	
6.93	0.04	Cytochrome P450 2K1-like	429	C:nucleus; F:DNA binding; P:cytoskeleton organization; F:protein heterodimerization activity; F:calcium ion binding; C:nucleosome; F:calcium-dependent phospholipid binding	

Fold change	padj	BLAST description	Length (bp)	GO Names list	
273.95	0.00	Melanoregulin-like protein	1131		Coloration
25.44	0.03	Melanocortin receptor binding NECAP endocytosis associated 2	2070	P:regulation of appetite; C:secretory granule; C:peroxisomal matrix; P:positive regulation of transcription from RNA polymerase II promoter; F:hormone activity; P:regulation of blood pressure; C:extracellular space; P:neuropeptide signaling pathway; P:glucose homeostasis; F:type 1 melanocortin receptor binding; P:cellular pigmentation; P:regulation of corticosterone secretion; F:type 3 melanocortin receptor binding; F:type 4 melanocortin receptor binding; P:regulation of glycogen metabolic process; P:negative regulation of tumor necrosis factor production	
12.71	0.05	Melanocortin m-phase phosphoprotein 8	2732	P:regulation of nucleobase-containing compound metabolic process; P:G-protein coupled receptor signaling pathway; P:single-multicellular organism process; F:peptide binding; F:melanocortin receptor activity; F:protein binding; P:cellular macromolecule metabolic process; F:carbohydrate derivative binding; C:intracellular membrane-bounded organelle; P:homeostatic process; C:membrane; P:regulation of gene expression; C:macromolecular complex	

13.52	0.00	Tyrosine kinase, non-receptor 2	1981	F:protein binding; C:proton-transporting ATP synthase complex; P:single-organism cellular process; P:nucleobase-containing compound metabolic process; P:transport; C:mitochondrial inner membrane; F:hydrolase activity	
